# Rice OsWRKY50 Mediates ABA-Dependent Seed Germination and Seedling Growth, and ABA-Independent Salt Stress Tolerance

**DOI:** 10.3390/ijms22168625

**Published:** 2021-08-11

**Authors:** Shuangzhan Huang, Lanjuan Hu, Shihan Zhang, Mingxing Zhang, Wenzhu Jiang, Tao Wu, Xinglin Du

**Affiliations:** Jilin Province Engineering Laboratory of Plant Genetic Improvement, College of Plant Science, Jilin University, Changchun 130062, China; huangsz@jlu.edu.cn (S.H.); hulj@jlu.edu.cn (L.H.); zsh971016@163.com (S.Z.); mingxing19@jlu.edu.cn (M.Z.); jwz1975@jlu.edu.cn (W.J.)

**Keywords:** ABA, OsWRKY50, rice, salt stress, WRKY transcription factor

## Abstract

Plant WRKY transcription factors play crucial roles in plant growth and development, as well as plant responses to biotic and abiotic stresses. In this study, we identified and characterized a WRKY transcription factor in rice, OsWRKY50. OsWRKY50 functions as a transcriptional repressor in the nucleus. The transcription of OsWRKY50 was repressed under salt stress conditions, but activated after abscisic acid (ABA) treatment. OsWRKY50-overexpression (OsWRKY50-OX) plants displayed increased tolerance to salt stress compared to wild type and control plants. The expression of *OsLEA3*, *OsRAB21*, *OsHKT1;5*, and *OsP5CS1* in OsWRKY50-OX were much higher than wild type and control plants under salt stress. Furthermore, OsWRKY50-OX displayed hyposensitivity to ABA-regulated seed germination and seedling establishment. The protoplast-based transient expression system and yeast hybrid assay demonstrated that OsWRKY50 directly binds to the promoter of *OsNCED5*, and thus further inhibits its transcription. Taken together, our results demonstrate that rice transcription repressor OsWRKY50 mediates ABA-dependent seed germination and seedling growth and enhances salt stress tolerance via an ABA-independent pathway.

## 1. Introduction

Plant transcription factors (TFs) encoded by WRKY genes are related to the regulation of various biological processes. These transcription factors are known as WRKY because of the highly conserved WRKYGQK structural domain of their N-terminals [1]. WRKY TFs are one of the largest transcription factor families, which have been reported to be involved in the regulation of many aspects of a plant’s lifecycle. Based on searches in the Plant Transcription Factor Database (PlantTFDB), there are 128 WRKY genes in *Oryza sativa* subsp. *Japonica* and 90 in *Arabidopsis thaliana* [2].

Many WRKY TFs have been identified and are regarded as significant regulators of various mechanisms, such as stress responses. Transgenic rice plants that overexpress OsWRKY51 show resistance to *Xanthomonas oryzae pv. oryzae* (Xoo) by means of activating the transcription of defense-related genes [3]. In addition, WRKY TFs also play key roles in plant growth and development. The rice dwarf and late flowering (*dlf1*) gene, which encodes an OsWRKY11 transcription factor, has influences on the agronomic traits of rice, such as plant height and flowering time [4]. The expression of *RAV1* is down regulated by AtWRKY6, and thus the transcription of *ABI3*, *ABI4*, and *ABI5*, which was repressed by RAV1, was activated in WRKY6 overexpression lines, which leads to ABA hypersensitivity phenotypes during *Arabidopsis* seed germination and post-germination seedling growth [5]. Furthermore, some WRKYs are multifunctional transcription factors that participate in both biotic and abiotic stress responses. Rice OsWRKY11 could directly bind to the promoter of defense gene *CHITINASE 2* and drought-responsive gene *RAB21* and activate their transcription. Therefore OsWRKY11 overexpression lines obtain enhanced resistance to bacterial pathogen *Xoo* and tolerance to drought stress [6].

WRKY TFs usually contain one or more WRKY domains, which is a sixty amino acids-long DNA binding domain (DBD) that can specifically recognize and bind a W-box ((T) TGACC (A/T)) in the prompters of target genes [7]. WRKY proteins could activate or inhibit the transcription of downstream genes by targeting to a W-box within their promoters. *Arabidopsis* AtWRKY63 was found to be able to bind to the W-box of ABA-responsive gene *ABF2* promoter and positively regulate *ABF2* transcription, which may result in the drought sensitivity phenotype of *atwrky63* mutant [8]. The expression of 1-aminocyclopropane-1-carboxylic acid synthase (ACS) genes were repressed by TaWRKY51 through binding to the W-box of *ACS2* promoter in wheat TaWRKY51 overexpression lines, which results in less ethylene production, thus producing more lateral roots [9].

Salt stress is one of major environmental factors that reduces cultivated land area, limits plant productivity, and threatens food security [10]. Unravelling the molecular mechanism of plant response to salt stress would be beneficial for improving crop salt tolerance [11]. It was reported that transgenic rice overexpressing Late embryogenesis abundant gene *OsLEA3-2* conferred resistance to salt stress [12]. OsHKT1;5 transporters were found to be involved in xylem Na^+^ unloading in leaf sheaths and the mediation of Na^+^ exclusion in the phloem to prevent Na^+^ transfer to young leaf blades [13]. Under salt stress, plants reduce the cell osmotic potential by accelerating the biosynthesis and accumulation of compatible osmolytes, such as proline, glucose, and glycine [11].

The phytohormone abscisic acid (ABA) is involved in signal transduction during plant growth and development and stress tolerance, such as seed dormancy, seed germination, stomatal closure, and abiotic stress response [14]. The components of ABA signaling transduction play vital roles in many aspects of plant lifecycles [15]. The accumulation of a positive regulator of ABA signaling, ABA Insensitive (ABI5), induces the expression of *PGIP* genes (*Polygalacturonase inhibiting protein*), which represses the activity of polygalacturanases and thus delays seed coat rupture and germination [16]. Environmental challenges, for instance, drought, salt, or cold stress, would induce the accumulation of ABA, which can activate the ABA signaling pathway in response to abiotic stresses. In the presence of ABA, protein kinase SnRK2.6 (OST1) was activated after dissociation from protein phosphatases type 2Cs (PP2Cs), and further regulates downstream gene *ABRE binding protein/ABRE-binding factors (AREB/ABFs)* to positively mediate osmotic tolerance [17]. On the other hand, plants also deal with osmotic stress via an ABA-independent pathway. The transcription factor DREB2 (DRE-/CRT-binding protein 2), which contains a *cis*-element dehydration-responsive element/C-repeat (DRE/CRT) in the promoter region, for instance *Arabidopsis DREB2A* and *DREB2B*, were induced by drought and salt stresses, but not by exogenous ABA [18,19]. Transgenic rice overexpressing *OsDREB1F* increases salt and drought tolerance via an ABA-independent pathway [20,21].

In this study, we identified a rice WRKY transcription factor OsWRKY50 and constructed OsWRKY50 overexpression lines. OsWRKY50-OX plants showed higher tolerance to salt stress compared to wild type (Kitaake, KT), while they displayed ABA hyposensitivity during rice seed germination and seedling growth.

## 2. Results

### 2.1. Expression Patterns of OsWRKY50

**Figure 1:**
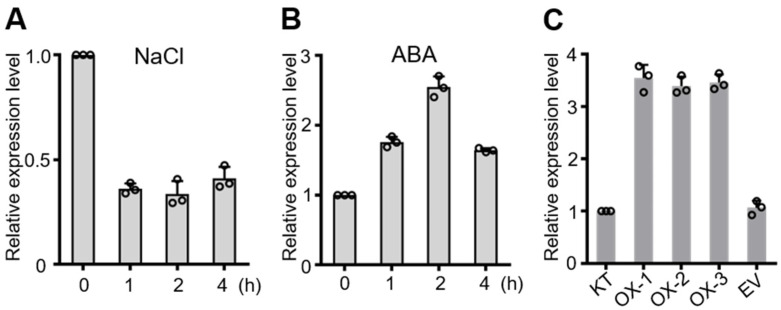
Expression patterns of OsWRKY50. (**A**,**B**) Transcript level of OsWRKY50 under salt stress (**A**) or ABA treatment (**B**) at 0, 1, 2, and 4 h. (**C**) Gene expression of OsWRKY50 in KT, overexpression lines, and EV plants. *Ubiquitin* was used as an internal control. Error bars indicate ± SD (*n* = 3).

Plant transcription factor WRKYs have been widely studied for their regulatory functions in plant growth and development and abiotic stress response. In this study, we found a member of the rice WRKY family, OsWRKY50, whose expression was influenced by salt stress. Two-week-old seedlings were exposed to 100 mM NaCl for the required time, and the RT-qPCR results show that OsWRKY50 transcription was repressed by salt stress (Figure 1A). Under high salinity conditions, the key plant hormone ABA is accumulated for stress-signaling transduction [22]. When treated with 20 μM ABA, the expression of OsWRKY50 was activated (Figure 1B). These results indicate that OsWRKY50 may be involved in salt stress and ABA responses. For the sake of elucidating the mechanism of OsWRKY50 in stress regulation, OsWRKY50 overexpression lines (OsWRKY50-OX) were generated. In addition, plants transformed with an empty vector (EV) were used as control plants (Figure 1C).

### 2.2. Characterization of OsWRKY50 Sequence and Subcellular Localization, and Phylogenetic Analysis

**Figure 2:**
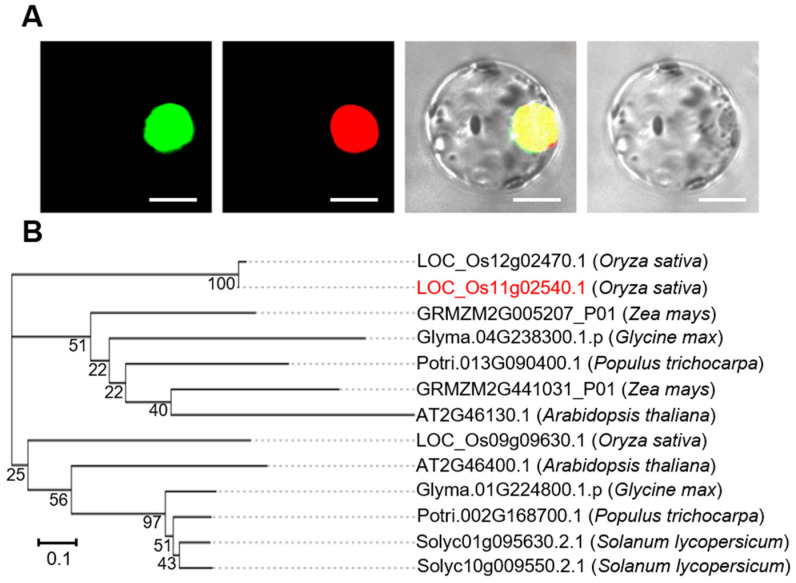
Subcellular localization and phylogenetic analysis of OsWRKY50. (**A**) Subcellular localization of OsWRKY50. The plasmid OsWRKY50*-GFP* was transformed into rice protoplasts together with *NLS-RFP* (nuclear localization signal fused with red fluorescent protein) and observed under a florescence microscope. The bars represent 10 µm. (**B**) Phylogenetic tree based on amino acid from each of the plant species. The phylogenetic tree was constructed by utilizing the MEGA5 Neighbor Joining algorithm based on conserved amino acid sequences calculated using the bootstrap method. Bootstrapping with 1000 replicates were shown at each node.

The rice genome annotation project database (http://rice.plantbiology.msu.edu, accessed on 22 April 2020) revealed that OsWRKY50 (LOC_Os11g02540) is located at chromosome 11, and has a nucleotide length of 990 bp, encoding 329 amino acids. The OsWRKY50 amino acid sequence was analyzed by SMART service, which showed that OsWRKY50 has a WRKY domain that is predicted to be located on the nucleus. In order to confirm the prediction, the OsWRKY50 coding sequence was fused to the 5′ end of a green fluorescent protein (GFP) to generate an OsWRKY50-GFP fusion protein. The OsWRKY50-GFP construct was transformed into rice protoplasts together with a nuclear localization signal (NLS-RFP) construct. The results showed that OsWRKY50-GFP is located on the nucleus, confirming that OsWRKY50 is a nuclear protein (Figure 2A). A phylogenetic analysis was performed for the sake of elucidating the evolutionary genetic relationships between OsWRKY50 and other WRKY proteins in plants, such as *Arabidopsis thaliana*, *Glycine max*, and *Zea mays*. As displayed in Figure 2B, OsWRKY50 showed low homology with other plant species; however, it showed high similarity with LOC_Os12g02470 (OsWRKY65) in *Oryza sativa* subsp. *japonica*.

### 2.3. OsWRKY50-OX Plants Showed Salt Stress Tolerance Phenotype

**Figure 3:**
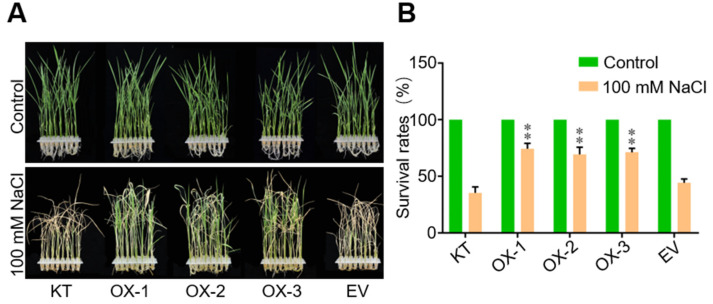
Phenotypes of KT, OX-1, OX-2, OX-3, and EV plants. (**A**) Two-week-old rice seedlings from KT, OX-1, OX-2, OX-3, and EV plants were treated with 100 mM NaCl for seven days and recovered for three days. (**B**) The survival rates under salt stress were measured according to (**A**). Error bars indicate ± SD (*n* = 3). ** *p*-value < 0.01 (Student’s *t*-test).

To further investigate whether OsWRKY50 is involved in salt stress response, rice wild type (Kitaake, KT), OsWRKY50-OX lines (OX-1, OX-2 and OX-3), and EV plants were subjected to 100 mM NaCl for seven days. The OsWRKY50-OX lines showed a similar phenotype to KT and EV plants under normal conditions; however, OsWRKY50-OX lines displayed more resistance to salt stress, showing higher survival rates than KT and EV after salt stress (Figure 3A,B). In conclusion, these results revealed that OsWRKY50 takes part in the salt stress response and may play a positive role in stress tolerance.

### 2.4. Overexpression of OsWRKY50 Modulates the Expression of Abiotic Stress-Responsive Genes under Salt Stress

**Figure 4:**
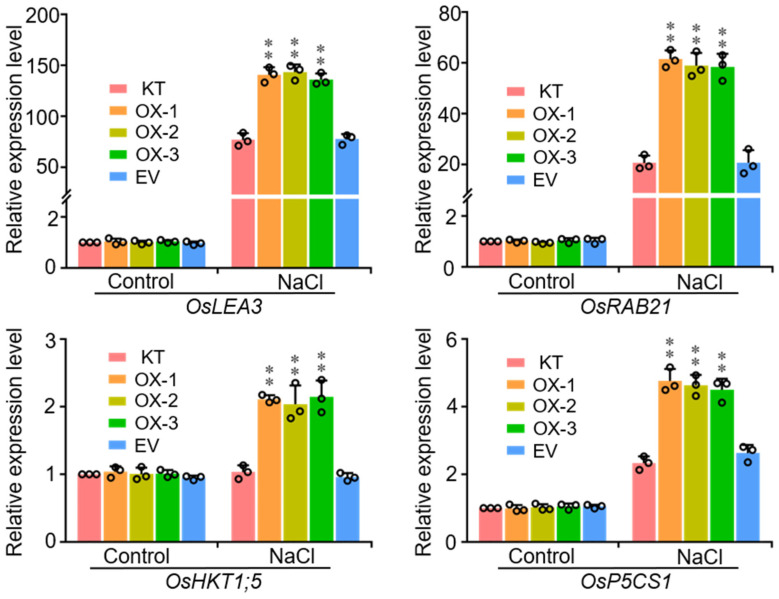
Expression levels of stress responsive genes. KT, OX-1, OX-2, OX-3, and EV plants treated with 100 mM NaCl for 1 h were collected for RNA extraction. Total RNA (2 μg) was used for cDNA synthesis by reverse transcription-polymerase chain reaction (RT-PCR). *Ubiquitin* was used as the internal control. Data represent means ± SD (*n* = 3). ** *p* < 0.01 (Student’s *t*-test).

To investigate the mechanism underpinning the salt stress tolerance of OsWRKY50-OX lines, two-week-old seedlings of KT, OX-1, OX-2, OX-3, and EV treated with 100 mM NaCl for 1 h were used to perform a RT-qPCR analysis. Several well-known stress-responsive genes, *OsLEA3* (Late embryogenesis abundant 3), *OsRAB21* (Responsive to ABA 21), *OsHKT1;5* (High affinity K^+^ transporter 1;5), and *OsP5CS1* (Pyrroline-5-carboxylate synthase 1), were selected for gene expression analysis. Under normal conditions, transcript abundances of *OsLEA3*, *OsRAB21*, *OsHKT1;5*, and *OsP5CS1* in OX lines did not show notable differences relative to KT and EV plants (Figure 4). However, the expression levels of these four genes were higher specifically in OX plants than in KT or EV plants under NaCl-treatment conditions (Figure 4), which demonstrated that OsWRKY50 positively regulates salt stress response by means of modulating the expression of abiotic stress-responsive genes.

### 2.5. OsWRKY50-OX Lines Showed ABA Hyposensitive Phenotypes

**Figure 5:**
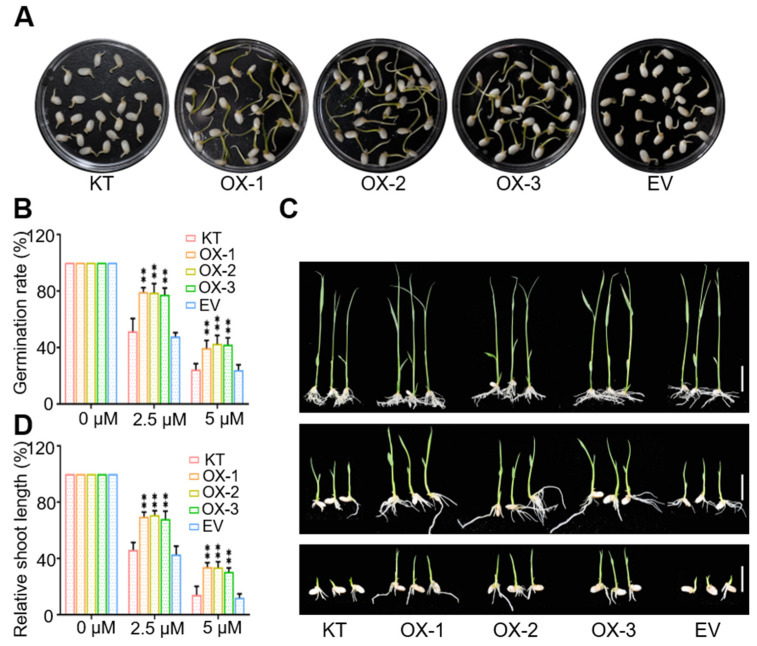
OsWRKY50-OX lines showed hyposensitivity to ABA response. (**A**) Germination phenotypes of KT, OX-1, OX-2, OX-3, and EV plant seeds. The seeds were germinated on MS medium containing DMSO (dimethyl sulfoxide), 2.5 µM, or 5 µM ABA. The seeds shown in (**A**) were supplemented with 2.5 µM ABA for five days. (**B**) Germination rates were measured in accordance with cotyledon greening emergences as shown in (**A**). (**C**) Phenotypes of rice seedlings treated with exogenous ABA. Two-day-old germinated seeds were transferred to MS medium containing 0 µM, 2.5 µM, or 5 µM ABA for four days. The bars represent 2 cm. (**D**) Statistical analysis of the relative shoot length under ABA treatment. Error bars indicate ± SD (*n* = 3). Three biological replicates were performed with 20 plants in each genotype. ** *p* < 0.01 (Student’s *t*-test).

It was reported that phytohormone ABA acts as a key endogenous messenger in response to abiotic stresses, such as drought and salt stress [23]. Next, we set out to determine the ABA sensitivity of OsWRKY50-OX lines during seed germination and seedling establishment. KT, OX-1–3, and EV seeds were incubated on Murashige and Skoog (MS) medium supplemented with DMSO (control) or different concentrations of ABA. The seed germination rates of OX-1–3 were much higher than those of KT and EV plants, confirming the ABA hyposensitivity of OsWRKY50-OX lines in seed germination (Figure 5A,B). In addition, two-day-old-seedlings were moved to MS medium containing 2.5 µM or 5 µM ABA for four days. As shown in Figure 5C,D, OX-1–3 displayed longer shoot length compared to those of KT and EV plants. In summary, the results proved that OsWRKY50 negatively regulates ABA-mediated seed germination and seedling establishment.

### 2.6. Overexpression of OsWRKY50 Affects ABA-Responsive Gene Transcription

**Figure 6:**
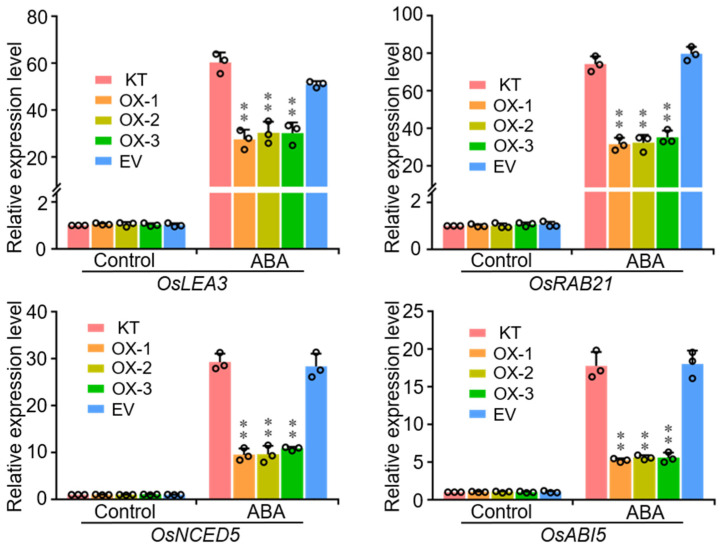
RT-qPCR analyses of ABA responsive genes. RNA was isolated from KT, OX-1, OX-2, OX-3, and EV treated with 20 μM ABA for 2 h. The expression of genes in KT under normal conditions are set to 1. *Ubiquitin* was used as the internal control. Data represent means ± SD (*n* = 3). ** *p* < 0.01 (Student’s *t*-test).

To further elucidate the regulatory function of OsWRKY50 in response to ABA, RT-qPCR was performed by using KT, OX-1–3, and EV seedlings treated with 20 μM ABA. Several ABA-responsive genes, such as *OsLEA3*, *OsRAB21*, *OsNCED5* (9-cis-epoxycarotenoid dioxygenase gene), and *OsABI5* (abscisic acid insensitive 5) were analyzed. The results showed that the expression levels of these stress-responsive genes were specifically lower in OsWRKY50 overexpression lines compared with KT and EV plants when treated with 20 μM ABA (Figure 6). Taken together, the special expression patterns of these stress-responsive genes may account for the ABA hyposensitive phenotypes of OsWRKY50 overexpression lines.

### 2.7. OsWRKY50 Protein Acts as a Transcriptional Repressor

**Figure 7:**
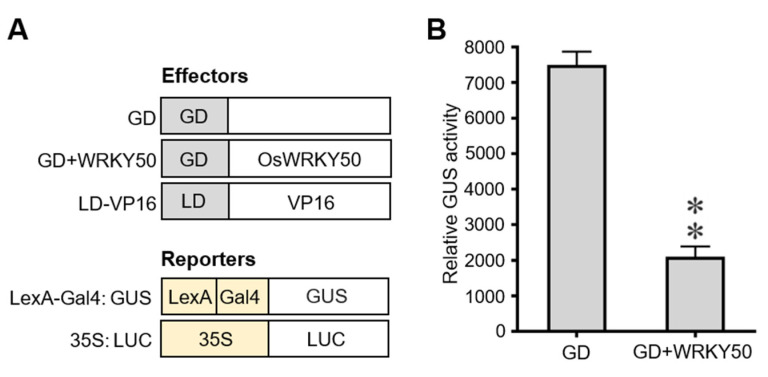
OsWRKY50 shows transcriptional repressor activity in vivo. (**A**) Schematic diagram of GAL4 DNA binding domain (GD)-OsWRKY50 and LexA DNA binding domain (LD)-VP16 fused proteins. (**B**) The relative GUS activity of protoplasts transformed with GD, LD-VP16, and LexA-Gal4:GUS or GD-WRKY50, LD-VP16, and LexA-Gal4:GUS. Protoplasts were isolated from two-week-old rice seedlings. The effector plasmid GD-OsWRKY50 or GD alone was co-transformed into rice protoplasts together with LD-VP16 and reporter plasmid LexA-Gal4: GUS. Plasmid 35S: LUC was used to normalize the transfection efficiency. Data represent the mean ± SD (*n* = 3). Statistical analyses were performed between GD and GD + WRKY50. ** *p* < 0.01 (Student’s *t*-test).

We have confirmed the transcriptional regulation of OsWRKY50 under salt stress and ABA responses, whether OsWRKY50 functions as a repressor or activator needs to be further investigated. Firstly, OsWRKY50 was fused with the GAL4-DNA binding domain and then transformed into yeast cells. The transformants of pBridge-OsWRKY50 and negative control (empty vector) failed to grow normally on double dropout media (SD/-His/-Trp). However, the positive control exhibited normal growth under the same conditions (Supplementary Figure S1). These results indicate that OsWRKY50 has no transcriptional activation in yeast cells. To investigate whether OsWRKY50 was a transcriptional repressor, the effector constructs GD (Gal4 DNA binding domain), GD-WRKY50, and LD-VP16 (LexA DNA-binding domain fused to the VP16 activation domain), together with reporter construct LexA-Gal4:GUS, were co-transformed into rice protoplasts using a PEG-mediated transformation system (Figure 7A). The reporter construct 35S:LUC was used as an internal control. As shown in Figure 7B, the relative GUS activity from protoplasts transformed by GD-WRKY50, LD-VP16, and LexA-Gal4:GUS was significantly repressed compared with that of protoplasts transformed by GD, LD-VP16, and LexA-Gal4:GUS. Overall, these results suggest that OsWRKY50 is a transcriptional repressor.

### 2.8. OsWRKY50 Directly Targets the Promoter of OsNCED5

**Figure 8:**
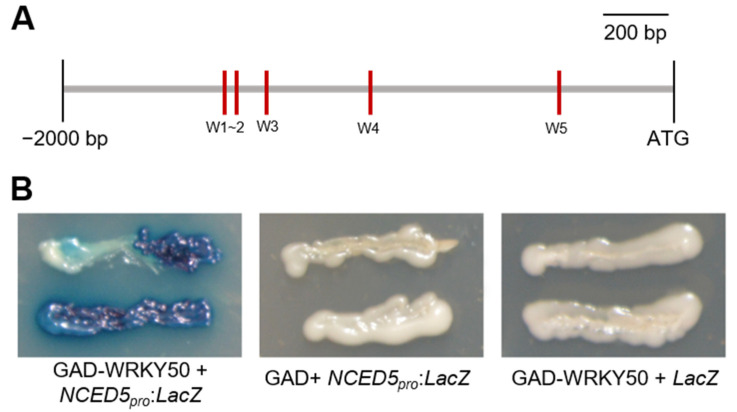
OsWRKY50 directly binds to the promoter of *OsNCED5*. (**A**) Schematic diagram of *OsNCED5* promoter. The W-box *cis*-elements (W1–W5) are shown in red boxes. Bar, 200 bp. (**B**) GAD-WRKY50 or GAD alone was transformed into the yeast strain EGY48 together with *OsNCED5_pro_:LacZ*. Subsequently, transformants cultured on SD/-Trp-Ura plates were transferred and grown on SD/-Trp-Ura medium containing X-gal at 30 °C for three days.

OsWRKY50 was predicted to contain a WRKY domain, which was reported to be indispensable for WRKY protein binding to the W-box (C/T) TGAC (T/C) in the promoter [24]. As OsWRKY50 functions as a transcriptional repressor, genes with lower expression were selected as potential direct downstream targets. Combined with the plant *cis*-acting regulatory elements database (PlantCARE), we found that the promoter of *OsNCED5* possesses five W-box sequences (Figure 8A). Furthermore, a yeast one-hybrid assay was performed to determine whether OsWRKY50 could directly bind to the promoter of *OsNCED5*. GAD-WRKY50 (GAL4 transcriptional activation domain-WRKY50 fusion proteins) or GAD alone, together with *OsNCED5_pro_: LacZ*, were cotransformed into yeast cells. Only GAD-WRKY50, but not GAD, could activate the *LacZ* reporter genes driven bythe *OsNCED5* promoter (Figure 8B). Together, these results showed that OsWRKY50 could directly bind to the *OsNCED5* promoter, and further repress its transcription.

## 3. Discussion

It has been reported that WRKY TFs are one of the largest transcription factor families in the plant kingdom [25]. In addition, they are involved in many aspects of transcriptional regulation networks, such as plant growth and development and biotic and abiotic stress responses. In this study, we identified a rice WRKY TF, OsWRKY50, which played a significant role in salt stress and ABA responses. We found that the expression of OsWRKY50 was repressed under salt stress and activated under ABA treatment (Figure 1). Furthermore, the promoter sequence of OsWRKY50 was analyzed by PlantCARE database. Some well-known *cis*-elements were within the OsWRKY50 promoter, such as MBS (MYB binding site), ABRE (ABA-responsive elements), TC-rich repeats, P-box (gibberellin-responsive element), and W-box (Supplementary Table S2), suggesting that OsWRKY50 takes part in abiotic stress and phytohormone responses and is probably regulated by other rice WRKY members.

According to a phylogenetic analysis, WRKY in other species showed low homology with OsWRKY50; however, OsWRKY65 (LOC_Os12g02470) amino acid sequences shared high similarity with OsWRKY50 under BLASTp analysis, especially in the WRKY domain amino acid sequence. Therefore, there may not be discernible phenotype differences between KT and single knock-out mutant *wrky50*, and the double mutant *wrky50 wrky65* may need to be constructed in order to further elucidate the molecular mechanism of OsWRKY50 in rice.

To confirm whether OsWRKY50 participates in abiotic stress, OsWRKY50-OX plants were generated and treated with 100 mM NaCl with KT and EV plants. The OsWRKY50-OX plants showed no distinguishable difference concerning phenotype compared to KT and EV under normal conditions. Notably, OsWRKY50-OX plants obtained special higher resistance to salt stress, which indicated that OsWRKY50 did take part in rice salt stress response. To further investigate the molecular mechanism underlying the salt stress resistance of OsWRKY50-OX plants, *OsLEA3*, *OsRAB21*, *OsHKT1;5*, and *OsP5CS1* were selected for RT-qPCR analyses. *LEA* genes such as *OsLEA3* and *OsRAB21* encode a late embryogenesis abundant protein, and overexpression of the *LEA3* gene could enhance osmotic tolerance in plants [26]. *OsP5CS1* encodes an enzyme pyrroline-5-carboxylate synthase (P5CS), which catalyzes the rate limiting reaction in proline biosynthesis in plants [27]. OsHKT1;5 transporters are involved in xylem Na^+^ unloading in leaf sheaths and mediating Na^+^ exclusion in the phloem to prevent Na^+^ transfer to young leaf blades [13]. Higher expression of these four stress-responsive genes in OsWRKY50-OX plants than that of KT could enhance salt tolerance under stress conditions.

In this study, we also found that OsWRKY50 participates in ABA response. *OsNCED5* encodes 9-cis epoxycarotenoid dioxygenase, which catalyzes a key step in the biosynthesis of an ABA precursor in plants. OsNCED5 increases ABA accumulation in rice and *OsNCED5* overexpressed in *Arabidopsis* showed delayed seed germination and repressed post-germination development [28]. ABI5 (Abscisic acid insensitive 5) could positively regulate ABA response as a transcription factor. Furthermore, overexpression of *OsABI5* in *Arabidopsis* leads to hypersensitivity in ABA-regulated seed germination [29]. The transcript levels of *OsNCED5* and *OsABI5* were lower in OsWRKY50-OX plants compared to KT and EV, which accounted for the ABA hyposensitive phenotypes of OsWRKY50-OX plants.

We have confirmed OsWRKY50 acts as a transcriptional repressor by utilizing the transient expression system using rice protoplasts. Notably, the expression of *OsLEA3*, *OsRAB21*, *OsHKT1;5*, and *OsP5CS1* in OsWRKY50-OX plants were much higher than those of KT and EV under salt stress condition, which illustrates that OsWRKY50 may influence the expression of these four genes via an indirect pathway. Furthermore, in this case, higher salt stress tolerance is not accompanied by hypersensitivity in ABA-regulated seed germination and seedling establishment, which was similar to previously reported results [8,30]. In addition, some genes from the ABA biosynthesis pathway were selected for RT-qPCR analysis, and the expression of *OsNCED2* and *OsABA2* in OsWRKY50-OX plants showed no difference from KT and EV under salt stress. Moreover, the expression of ABA biosynthesis gene (*OsNCED5*) in OsWRKY50-OX plants was much lower than that of KT and EV under salt stress (Supplementary Figure S2). Furthermore, OsDREB1F was reported to increase rice salt tolerance via an ABA-independent pathway. We found that the expression of *OsDREB1F* in OsWRKY50-OX plants was much higher than that in KT and EV (Supplementary Figure S2), which indicates that OsWRKY50 may regulate salt stress tolerance independent of the ABA signaling pathway. Combined with the yeast one hybrid system, we conclude that OsWRKY50 directly targets *OsNCED5* and thus represses its transcription. OsWRKY50 appears to have a positive role in salt stress response by activating the expression of stress-responsive genes in an ABA-independent manner. However, it also plays a negative role in ABA-regulated seed germination and seedling establishment by inhibiting the expression of ABA biosynthesis gene *OsNCED5*.

Taken together, we identified a nuclear-localized transcription factor, OsWRKY50, that negatively regulates the ABA signaling pathway by directly binding to the promoter of *OsNCED5* and repressing its transcription. In addition, OsWRKY50 may positively regulate salt stress response via an ABA-independent signaling pathway.

## 4. Materials and Methods

### 4.1. Plant Growth Condition and Transgenic Plant Generation

*Oryzasativa* ssp. *japonica* variety Kitaake (KT) was regarded as a wild type plant in this study. The rice seeds were sterilized with sodium hypochlorite solution for at least 30 min, then were rinsed more than five times using sterile distilled water. The seeds were germinated in water for three days at 37 °C. Rice seedlings were grown in hydroponic culture and put in a growth chamber with a temperature of 28 °C during the day and 25 °C at night and with 50% humidity. The *pUbi1390*-OsWRKY50 construct was transformed to rice calli by an *Agrobacterium tumefaciens*-mediated transformation. Next, fifteen positive transgenic lines were obtained using Sanger sequencing. T_2_ homozygous seedlings were used in this study.

### 4.2. Plasmid Construction

OsWRKY50 cDNAs were isolated and obtained from rice cDNA library by PCR using OsWRKY50-specific primers. To generate the *OsNCED5_pro_:LacZ* construct, the 2000 bp promoter fragment of *OsNCED5* was amplified from rice genomic DNA, then the fragment was inserted into pLacZi-2μ by homologous recombination. To generate the *pJG4-5-*OsWRKY50 construct, OsWRKY50 *CDS* was cloned into pJG4-5 by homologous recombination. To generate the pBridge-OsWRKY50 construct, the OsWRKY50 CDS fragment was cloned into pBridge by homologous recombination. All primers are listed in Supplementary Table S1.

### 4.3. Salt Stress Treatment and ABA Sensitivity Test

Two-week-old seedlings of KT and OsWRKY50-OX were cultured in hydroponic culture solution containing 100 mM NaCl for seven days [31]. Subsequently the seedlings were transferred to normal culture solution for another three days and the survival rates were measured. To test the sensitivity of ABA-regulated seed germination, sterilized rice seeds were germinated on MS medium containing 0 µM, 2.5 µM, or 5 µM ABA for four days and germination rates were measured. In addition, to test the ABA sensitivity on seedling development, seeds of different genotypes were germinated on MS medium for two days and transferred to MS solid medium containing 0 µM, 2.5 µM, or 5 µM ABA. The plates were put in a plant incubator with temperature 28 °C/25 °C (day/night) and photoperiod 14 h/10 h (light/dark). After three days, shoot length was measured and used for calculating relative shoot length. The above-mentioned assays were repeated three times with 30 plants each.

### 4.4. Subcellular Localization and Phylogenetic Tree Construction

Rice protoplasts were isolated from 2-week-old seedlings grown on MS medium. The plasmids OsWRKY50-GFP and NLS-RFP were used for PEG-mediated protoplast transformation. After overnight culture, fluorescence microscope was used to observe GFP and RFP (nucleus localization) signals.

The phylogenic tree of OsWRKY50 was constructed on the basis of the bootstrap method (1000 replications) using MEGA5.0 software. Protein sequences used for phylogenic analysis are as follows: LOC_Os11g02540.1, LOC_Os09g09630.1, and LOC_Os12g02470.1 (*Oryza sativa*); AT2G46130.1 and AT2G46400.1 (*Arabidopsis thaliana*); Glyma.01G224800.1.p and Glyma.04G238300.1.p (*Glycine max*); GRMZM2G441031_P01 and GRMZM2G005207_P01 (*Zea mays*); Potri.002G168700.1 and Potri.013G090400.1 (*Populus trichocarpa*); Solyc01g095630.2.1 and Solyc10g009550.2.1 (*Solanum lycopersicum*).

### 4.5. RNA Isolation and RT-qPCR

Rice seedlings treated with 100 mM NaCl or 20 µM ABA were harvested into liquid nitrogen at the indicated time. High-quality RNA from seedling tissues was extracted and purified using a TRIzol-based procedure, then two micrograms RNA was reverse-transcribed to cDNA by using cDNA Synthesis SuperMix Kit (TransGen, Beijing, China). Quantitative real-time polymerase chain reaction (RT-qPCR) was performed under ABI QuantStudio Real-Time PCR System using SYBR Green-based PCR assay. The results of RT-qPCR were analyzed by the 2^−ΔΔCt^ method, and the rice *ubiquitin* gene was used as an inner reference. The statistical significance was evaluated according to Student’s *t*-test analysis. Primers used in RT-qPCR analysis can be found in Supplementary Table S1.

### 4.6. Transactivation Activity and Yeast One-Hybrid (Y1H) Assays

TA transactivation activity assay was performed according to the manufacturer’s protocol (Clontech). The plasmid pBridge-OsWRKY50 was transformed into the yeast strain Y2HGold, in addition, pBridge-OsRPH1 was used as a positive control [32], and the empty vector *pBridge* was used as a negative control. Then, the transformants grown on a single-dropout media (SD/-Trp) were subcultured on double-dropout media (SD/-His/-Trp).

The procedure of Y1H was modified slightly based on a previous study [33]. *pJG4-5-OsWRKY50* or *pJG4-5* empty vector was transformed into the yeast strain EGY48 together with *OsNCED5_pro_:LacZ*, then transformants were grown on SD/-Trp-Ura plates at 30 °C. After three days the positive clones were subcultured on SD/-Trp-Ura medium supplemented with 5-bromo-4-chloro-3-indolyl-beta-d-galactopyranoside (X-gal) at 30 °C and X-gal staining was checked after three days.

### 4.7. Transcription Inhibition Assay

Transcription activity was performed using a protoplast transient expression system as described previously with slight modifications [34]. Protoplasts were isolated from two-week-old rice seedlings grown on MS medium. The effector plasmid GD-OsWRKY50 or GD (Gal4 DNA binding domain) alone was co-transformed into rice protoplasts together with LD-VP16 and reporter plasmid LexA-Gal4: GUS. Plasmid 35S: LUC was used to normalize the transfection efficiency. The transformed protoplasts were incubated at 24 °C for 12 h, and luciferase and GUS activities were measured, respectively. The transcription activity was measured according to the ratio of GUS activity relative to LUC activity (GUS/LUC).

## Supplementary Materials

The following are available online at https://www.mdpi.com/article/10.3390/ijms22168625/s1.
