# Supplementary material for: Rice OsWRKY50 Mediates ABA-Dependent Seed Germination and Seedling Growth, and ABA-Independent Salt Stress Tolerance
Source: Int J Mol Sci. 2021 Aug 11;22(16):8625. doi: 10.3390/ijms22168625 (PMC8395310; doi:10.3390/ijms22168625)
Supplement: Supplementary file 1 [file ijms-22-08625-s001.zip › OsWRKY50-Supplementary Figures.pdf]

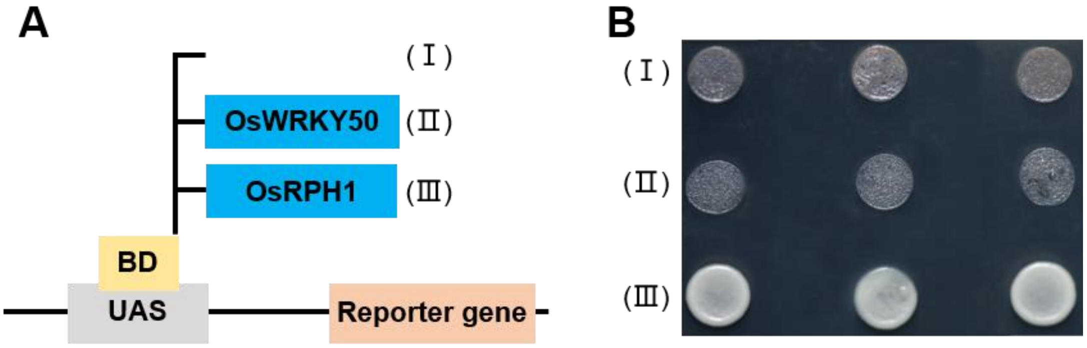

Supplementary Figure S1. OsWRKY50 has no transcriptional activation activity in yeast cells. (**A**) Schematic diagram of recombinant plasmids. GAL4 DNA-binding domain (BD) **-** OsWRKY50 (II), BD alone (I) as a negative control, or BD-OsRPH1 used as a positive control (III), were expressed in Y2HGold yeast strain. UAS: upstream activating sequence. (**B**) Y2HGold yeast strain containing fused protein were cultured on SD/-His/-Trp medium. BD alone (I) as a negative control, BD-OsRPH1 (III) as a positive control.

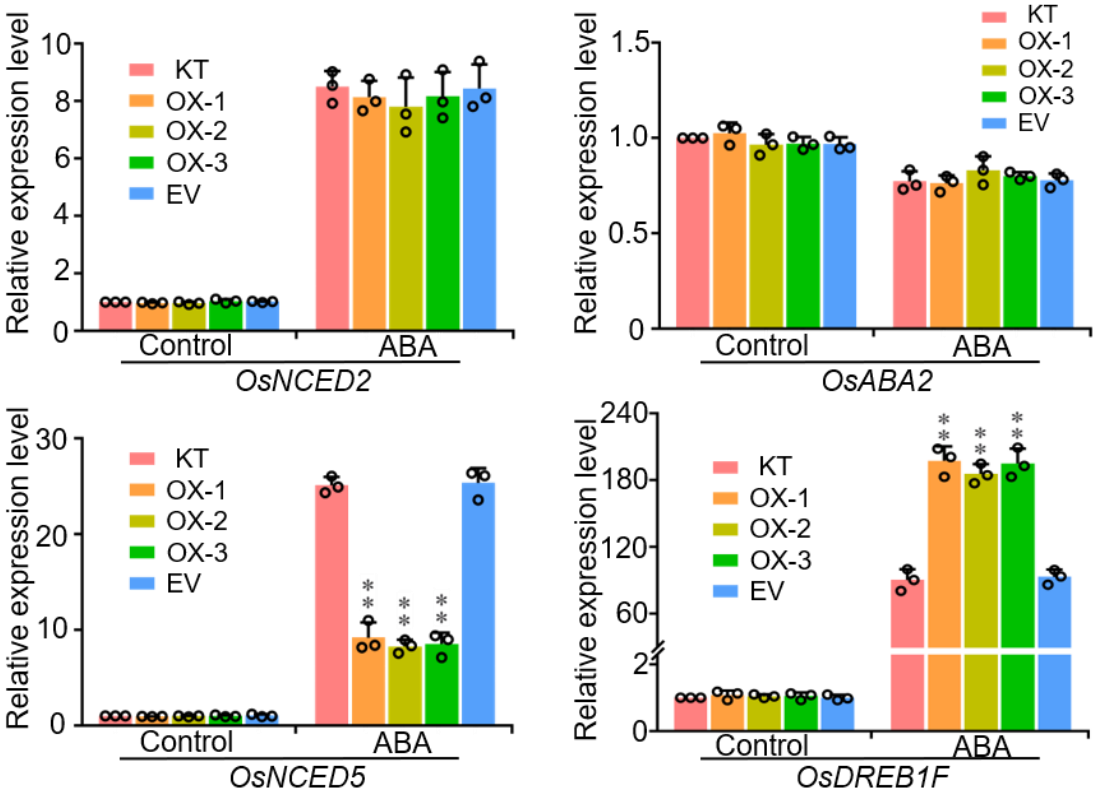

Supplementary Figure S2. RT-qPCR analysis of responsive genes under salt stress. KT, OX-1~3, and EV plant materials were collected when treated with 100 mM NaCl for 1 h. *Ubiquitin* was used as the internal control. Data represent means ± SD (n = 3). **, $P < 0.01$ (Student's t-test).
